# Embed capacity development within all global health research

**DOI:** 10.1136/bmjgh-2020-004692

**Published:** 2021-02-17

**Authors:** Ayola Akim Adegnika, John H Amuasi, Paulin Basinga, Della Berhanu, Araya Abrha Medhanyie, Yemisrach Behailu Okwaraji, Lars Åke Persson, Bonaventure Savadogo, Joanna Schellenberg, Peter Steinmann

**Affiliations:** 1 Centre de Recherches Médicales de Lambaréné, Lambaréné, Gabon; 2 School of Public Health, Kwame Nkrumah University of Science and Technology, Kumasi, Ghana; 3 Bill & Melinda Gates Foundation, Seattle, Washington, USA; 4 London School of Hygiene & Tropical Medicine, London, UK; 5 MARCH Research Centre and School of Public Health, Mekelle University, Mekelle, Tigray, Ethiopia; 6 Swiss Tropical and Public Health Institute, Basel, Basel-Stadt, Switzerland

**Keywords:** child health, control strategies, public health

**Summary box**

- All countries, including low-income and middle-income countries, need robust health research capacity.
- The research capacity gap between the Global North and Global South is closing too slowly, and governments, funders and academic institutions are not investing sufficiently to bridge this chasm.
- With two examples from collaborative research projects in sub-Saharan Africa, we illustrate how capacity development can be an integrated part of a joint research effort supported by all involved institutions.
- We advocate that research capacity development be valued as highly as evidence generation and be embedded in all global health research.

Global health research is expanding.^1^ New global health institutions appear in the North, sometimes by relabelling existing entities. Global health journals have been born, and funding schemes with this focus have increased. However, there are insufficient local competencies for research in low-income countries. The capabilities for problem-solving are growing too slowly to close the existing North–South capacity gap.^2^ Local problems and contexts are not sufficiently taken into account,^3^ and there is a continued dependency on institutions in the Global North.^4^ In this commentary, we aim to draw attention to the opportunity for capacity development within international collaboration for global health research. We illustrate this with examples of two collaborative projects in sub-Saharan Africa, where mutual capacity development was embedded as an integral part of the project.

A review of health research capacity development identified a range of strategies but a lack of robust evaluation that resulted in unclear effectiveness and weak learning.^5^ Only a few global health funders systematically allocate resources to research capacity development.^6^ Power relations between collaborating partners from the Global North and Global South continue to lead to unfair ownership of data, unfair authorship and other problems that together have been labelled a neocolonialist model of capacity building.^6^

Countries in sub-Saharan Africa allocate few resources to develop research capacity.^7^ Further, academic institutions’ research culture in the Global South is often theoretical and even detached from the health system challenges in their own countries.^8^ With the COVID-19 pandemic, it is evident that low-income and middle-income countries have a great need for robust research and implementation capacity, which is essential for monitoring, managing and overcoming the current challenges. There is a risk that previous funding to develop such ability is disrupted by the pandemic crisis.^9^ Instead, it should act as a spur to reinforce research capacity development in the Global South.

During the current pandemic, we have been reminded that examples of good public health management may be found in the Global South, in sharp contrast to some of the North.^10^ The notion of what has been labelled reverse innovation is not new. There are many examples of innovations created in the South that have provided chances for shared learning to the North. An equitable partnership in research offers opportunities for joint innovations and mutual capacity development.^11^

Our first case study comes from Ethiopia (box 1). Research capacity development was embedded within the evaluation of a community-based intervention in the four most populous regions. The Ministry of Health initiated this effort by creating a consortium that included four universities, the national public health institute, non-governmental organisations supporting programme implementation and an international academic partner. The funder earmarked resources for capacity development, including mentoring and support of the involved PhD students at the Ethiopian universities. The intention was to promote research training focused on ‘real world’ health system questions for doctoral students from the participating academic institutions and regional health bureaux. Students were registered at the Ethiopian universities and received supervision from the local and international partner universities. The selection of research topics for the different PhD studies balanced the intervention project’s evaluation needs and the individual students and supervisors’ research interests. Over time, all students and supervisors were convinced by the concept. They appreciated the broadened collaborating network across academic institutions and health system partners and enhanced health system and implementation science training opportunities.

Box 1 **Ethiopia. Implementation research with embedded capacity development**

Ethiopia successfully reduced child mortality during the past decades, but coverage, utilisation and quality of primary newborn and child health services have remained low. In 2016, the Ethiopian government initiated a complex intervention to enhance primary care for sick children. A barrier analysis and evidence synthesis informed the intervention, including community engagement activities, supportive supervision, training and mentoring of primary care workers and efforts to enhance district ownership and accountability for child health services. Four major non-governmental organisations coordinated the implementation of the intervention. With the vision to enhance national excellence and build capacity for health system research, the government created a consortium with four Ethiopian universities, the Ethiopian Public Health Institute, the non-governmental organisations supporting programme implementation and the London School of Hygiene & Tropical Medicine, with funding from the Bill & Melinda Gates Foundation. The consortium was tasked to evaluate the process and effectiveness of the intervention and embed capacity development at the local universities.^12^ Ten PhD students from the Ethiopian universities, including candidates from the regional health bureaux, were engaged in the 5-year evaluation. The students selected topics for their doctoral studies that were linked to the overall evaluation. For example, they studied equity in the utilisation of services,^13^ spatial analyses of care utilisation,^14^ quality of primary care and community leaders’ role in promoting the use of services. Academics from local universities and international partners formed supervisory teams, and the universities rotated in hosting workshops and courses of relevance for capacity development in implementation science.

Our second case study focuses on embedded research capacity development within a programme addressing neglected tropical diseases (NTDs) in the Central African Region (box 2). The project aimed to strengthen innovative NTD control and train a cadre of researchers, so that they became aware of implementation challenges and worked directly with key actors in NTD control and elimination. In the competitive selection of PhD candidates, countries with a weak university infrastructure suggested fewer qualified candidates, and there were considerable language barriers. It was a hurdle to navigate different administrative enrolment regulations. The selected candidates proposed a wide range of relevant research, all addressing priorities in their country’s NTD control programme. Opportunities to interact with public health stakeholders and tailored training courses were very well received. Regular supervision ensured that emerging issues were efficiently addressed, and stipends allowed the candidates to focus on their thesis work. Regular meetings were designed to foster contacts between candidates and establish connections with the existing African Research Network for Neglected Tropical Diseases.

Box 2 **Central African Region. Neglected tropical diseases control with embedded capacity development**

The Central African Region lags behind other parts of Africa in progress towards meeting the neglected tropical diseases (NTD) control and elimination targets.^15^ The 4-year project ‘Lutte contre les maladies tropicales négligées’ aims to sustainably strengthen capacity in the Communauté Economique et Monétaire de l'Afrique Centrale (CEMAC) region, including Cameroon, Chad, Central African Republic, Republic of Congo, Gabon and Equatorial Guinea. The project is implemented by the Organisation de Coordination pour la lutte contre les Endémies en Afrique Centrale (OCEAC) based in Yaoundé, Cameroon, with funding from the German Federal Ministry for Economic Cooperation and Development through the KfW Development Bank. The objectives are: (1) to support interventions to control or eliminate NTDs; and (2) to build local NTD research capacity through 19 PhD student fellowships. Supported intervention projects are aligned with national strategic plans. When completed, research projects are expected to support innovation and local or regional NTD control. Other features include: (1) priorities were defined by national strategic plans; (2) all NTDs, standard interventions and research disciplines were eligible; (3) proposals were entirely based on local priorities and could be submitted by national programmes, non-governmental organisations or consortia; (4) projects could focus on a specific target group, subnational region (including cross-boundary regions) or one or several countries; and (5) collaboration and exchange between researchers and control programme managers were encouraged. The aim is to train scientists who understand the NTD control needs of their home country. Students were enrolled at universities in their home country and benefited from cosupervision from partner universities. Research findings should enhance local NTD control and researchers who are embedded in the regional public health community.

These case studies provide examples of embedding PhD training into research efforts, where the scientific agenda is locally defined, bringing together academic institutions from different countries, and networking with the health system, non-governmental organisations and other stakeholders. Such environments may enhance scientific skills together with local and tacit knowledge. Structures and strategies are needed to manage conflicts and address challenges in such collaborations. Some funders, who would hesitate to embark on isolated long-term capacity development commitments, could be prepared to engage if the capacity development was embedded into the research performed by partnerships of universities, non-governmental organisations and the health system.

Multiple actions are needed to bridge the capacity gap in global health research. Academic institutions and researchers in the Global South and Global North, governments and global health research funders all have obligations to enhance equity in health research capacity.^6^ Funders have an essential role in promoting and including capacity development as an integrated part of global health research projects. Global North universities need to adjust their promotion policies to enhance equity in global health partnerships.^4^ Academic institutions in low-income and middle-income countries should prioritise developing local competencies in their health research projects. Governments in the Global South should allocate more resources into research capacity as a vital development investment. We propose that research capacity development be valued as highly as the generation of new scientific knowledge—by the academicians and universities in the Global South and Global North, by global health research funders and by governments.
